# Lost Denture Found in Esophagus After a Decade: A Rare Case Report

**DOI:** 10.7759/cureus.8042

**Published:** 2020-05-09

**Authors:** Raveena Karthikeyan, Chandramohan S M, Sakthivel Harikrishnan, Vigneshwaran VB, Balaji Singh

**Affiliations:** 1 Surgery, Madras Medical College, Chennai, IND; 2 Surgery, Sri Ramachandra Institute of Higher Education and Research, Chennai, IND; 3 Surgery, ESOINDIA - Centre for Gastro Esophageal Disorders, Chennai, IND; 4 Surgical Gastroenterology and Liver Transplant, Government Stanley Medical College, Chennai, IND; 5 General Surgery, Sri Ramachandra Institute of Higher Education and Research, Chennai, IND

**Keywords:** esophageal foreign body, denture, thoracotomy, intercostal muscle flap

## Abstract

Dentures are accidentally ingested foreign bodies, especially in the geriatric population. They get frequently lodged in the esophagus because of their larger size, rigidity, and pointed edges. But, it is unusual for a denture to remain asymptomatic in the esophagus for a decade. We report a case of 45-year-old female who presented with the complaints of progressive dysphagia for six months. Endoscopy revealed an impacted denture in the mid-esophagus. The patient recollected that she lost her denture 13 years back and was unaware that she swallowed it. Right thoracotomy and esophagotomy were done to remove the impacted denture. The esophagotomy site was buttressed with vascularised intercostal muscle flap.

## Introduction

Accidental ingestion of foreign bodies is commonly seen in the extremes of age group (children and elderly). Dentures, meat boluses, and fish bones are the most commonly ingested foreign bodies in the elderly population [1]. Once a foreign body has passed beyond the cricopharynx, it frequently gets lodged in the esophagus because it has weak peristalsis and multiple anatomical narrowings [1,2]. Longstanding impaction might lead to mucosal ulceration, perforation, sepsis, and death [2]. This article reports an interesting case of the impacted denture in the esophagus of a 45-year-old female for more than a decade. This case is unique because of the duration for which the denture remained asymptomatic in the esophagus and for its successful surgical management.

## Case presentation

**Figure 1:**
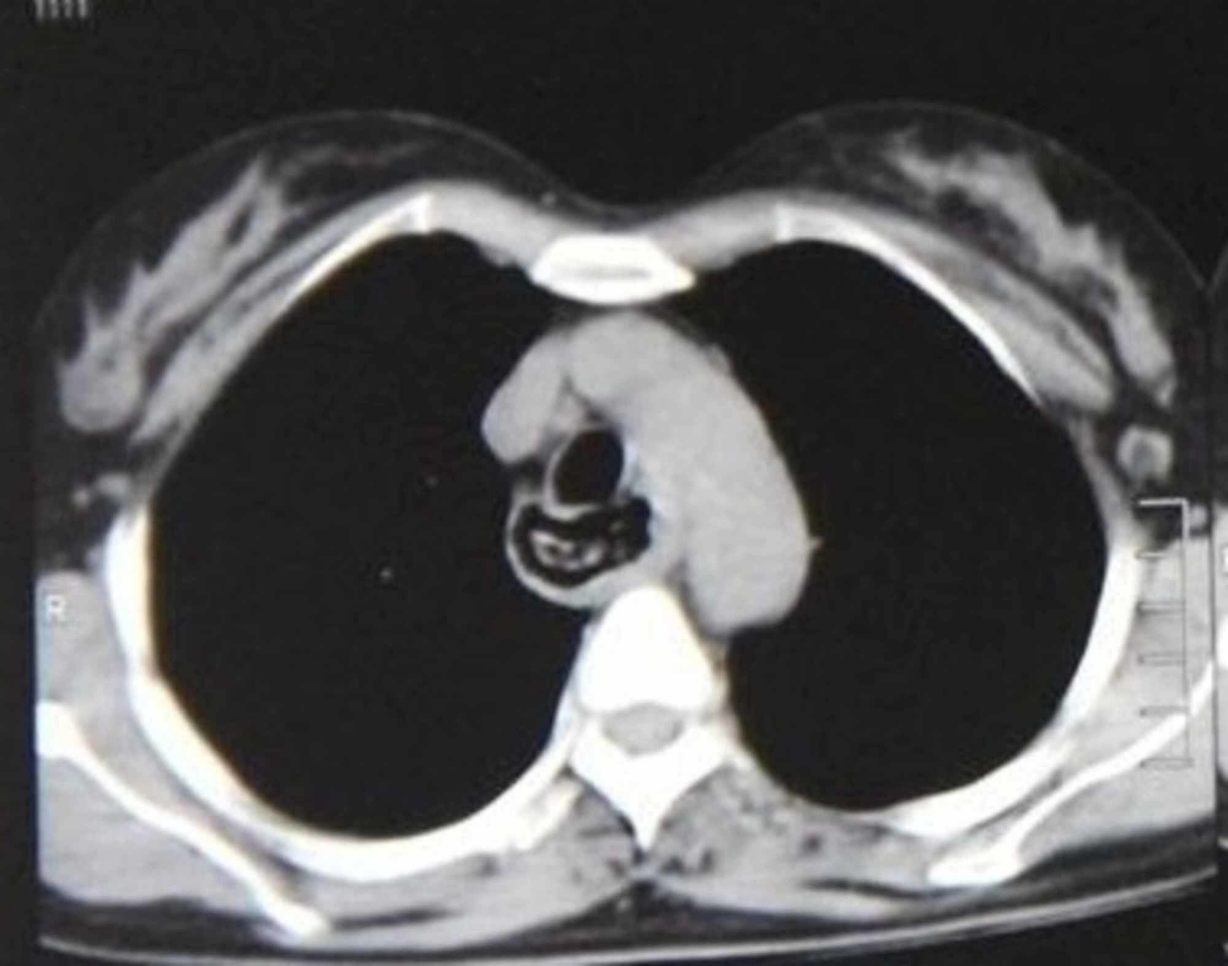
CT scan showing crescent-shaped foreign body in the esophagus

A 45-year-old female presented with complaints of progressive dysphagia for six months. Outside endoscopy showed a friable and fixed lesion in the esophagus suspicious of malignant growth and biopsy was taken. She was referred to us after the biopsy came out to be negative. We did a repeat endoscopy that revealed an impacted denture in the mid-esophagus. On eliciting the history, the patient recollected that she lost her denture 13 years back and was unaware that she swallowed it. On examination, the patient was hemodynamically stable. X-ray and CT scan revealed a crescent-shaped hyperdense foreign body in the esophagus at the level of aortic arch T4 (Figure 1).

**Figure 2:**
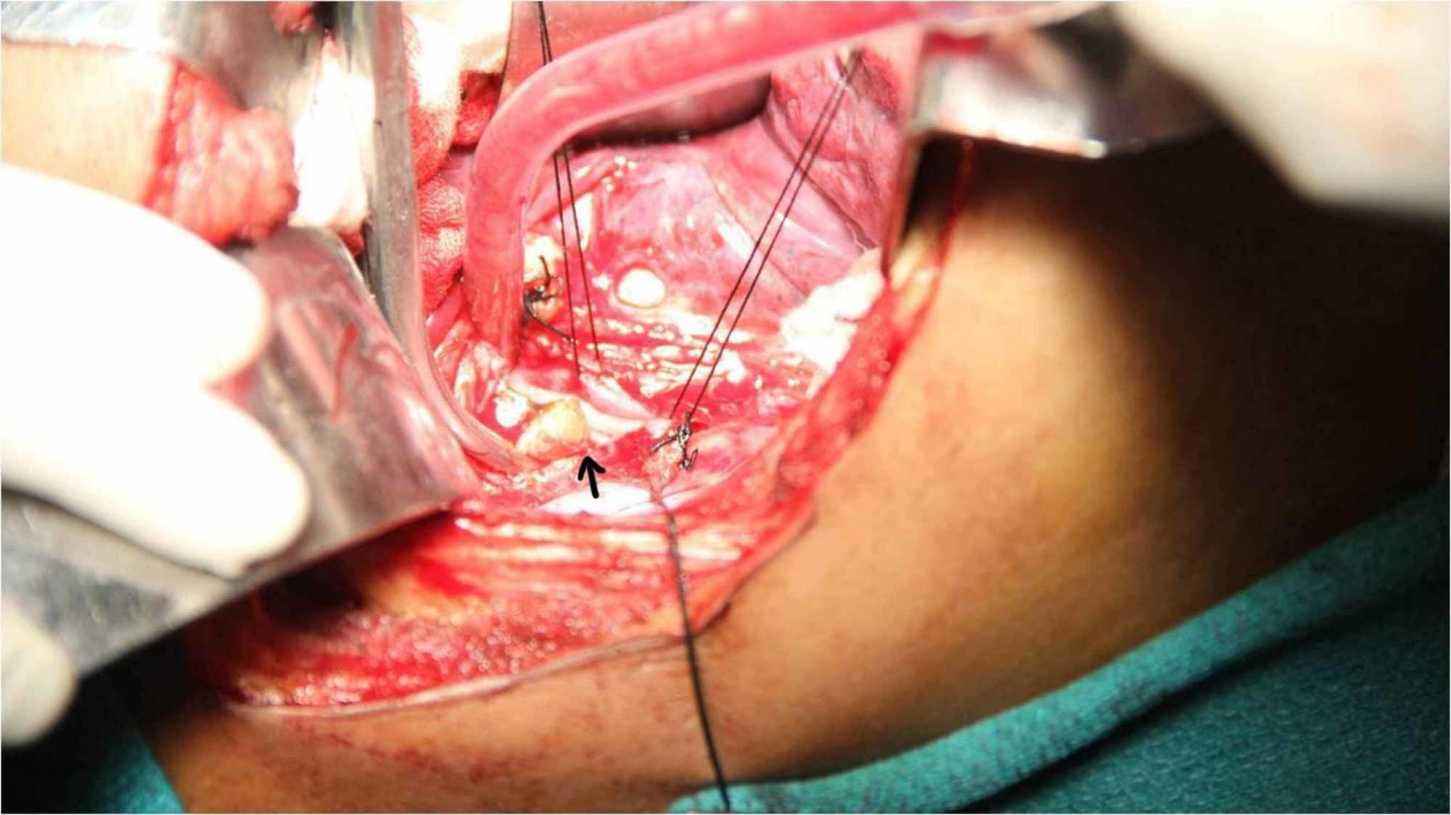
Removal of denture through the esophagotomy site (arrow)

**Figure 3:**
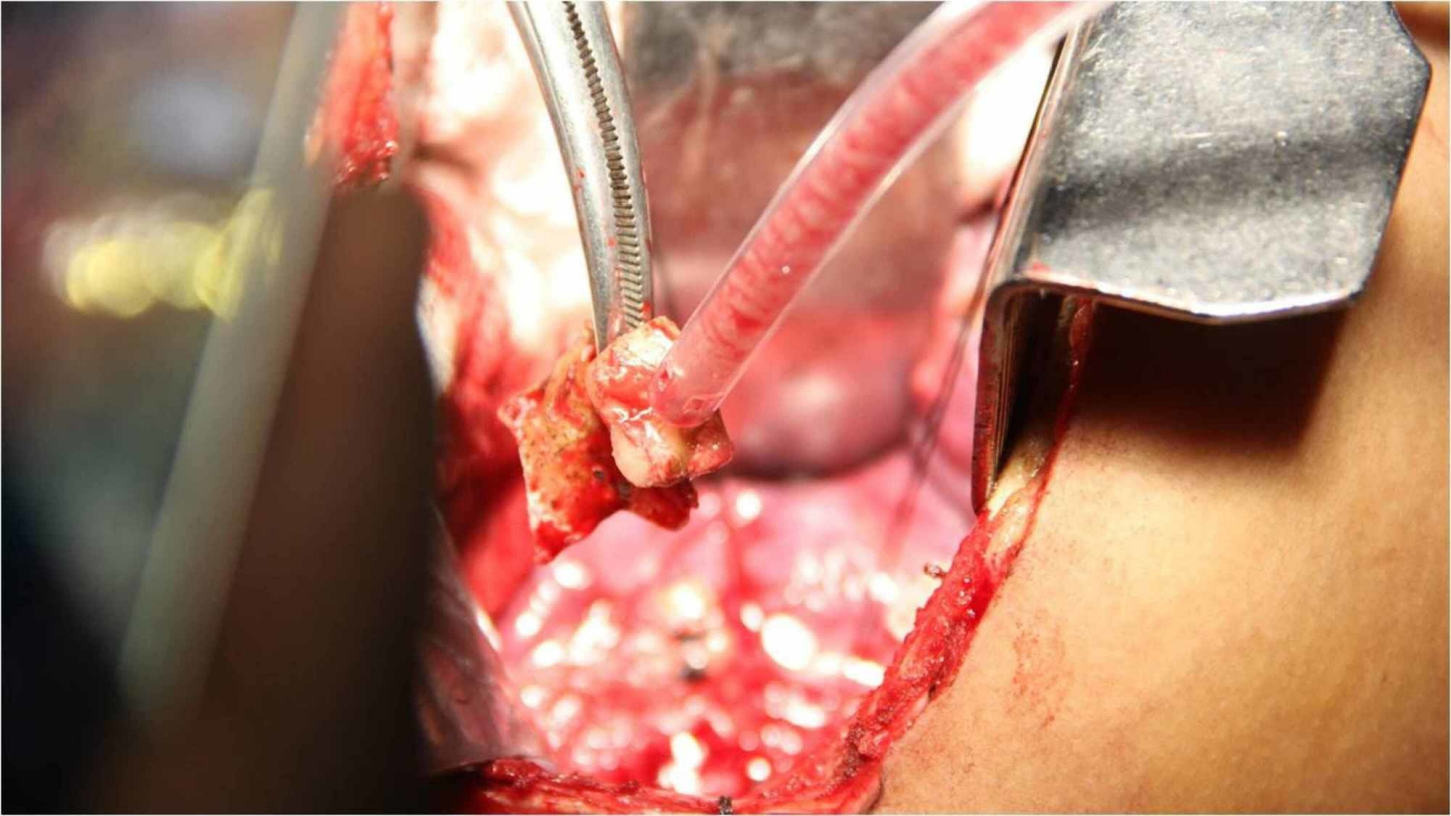
Intra-operative picture showing removed denture

Under general anesthesia, the patient was positioned in left lateral side and thoracotomy was made in the right fifth intercostal space. The impacted denture was identified and removed by esophagotomy (Figures 2-3).

**Figure 4:**
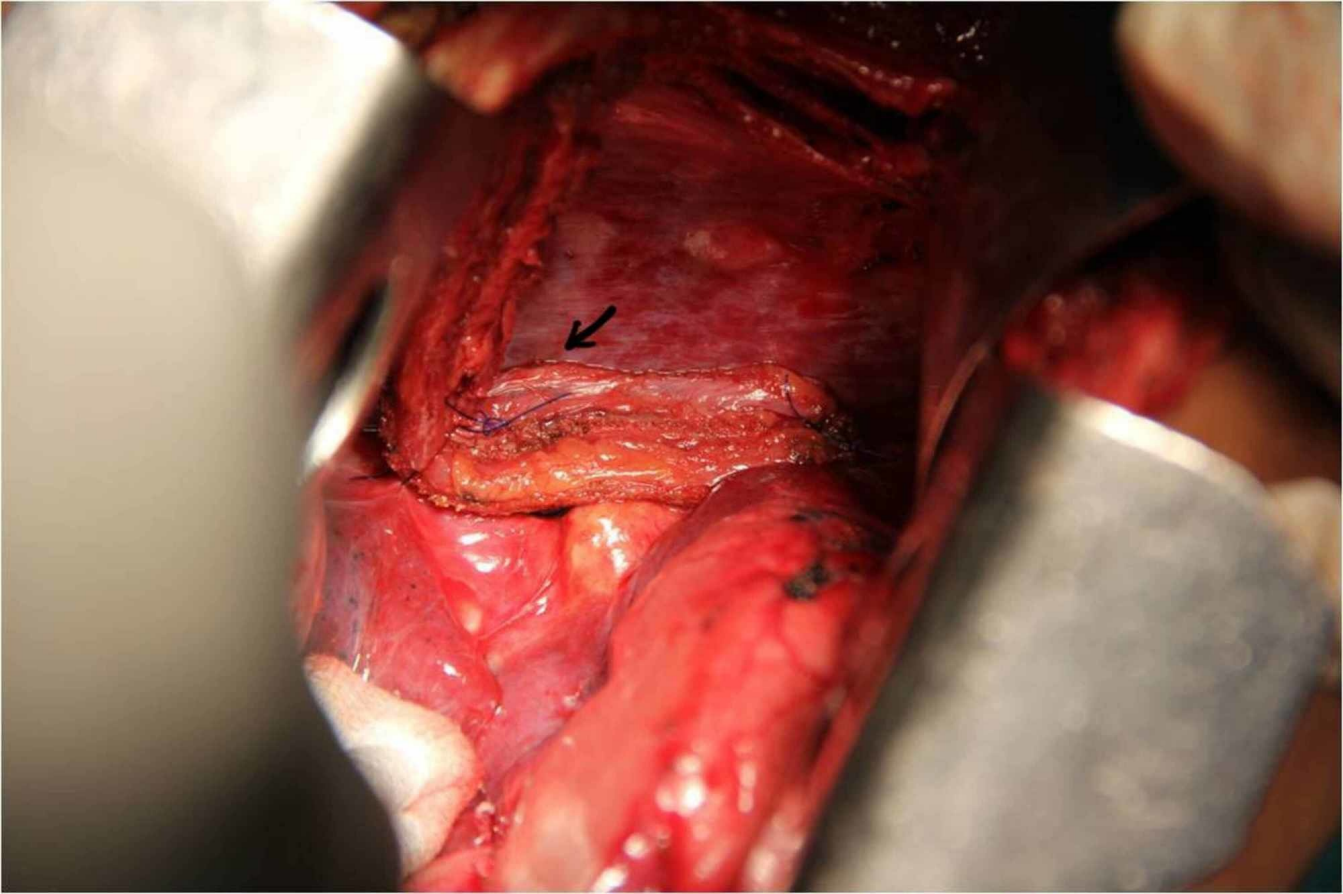
Esophagotomy site is reinforced with intercostal muscle flap (arrow)

The esophagotomy site was closed with interrupted 3-0 polydioxanone (PDS) sutures and reinforced with viable pedicled muscle flap from right fifth intercostal space (Figure 4).

The patient had an uneventful postoperative recovery. Oral intake was started on postoperative day three and the patient was discharged on postoperative day seven.

## Discussion

Accidental ingestion of a foreign body is common in both children and adults. In adults, it is mostly observed in the elderly population and psychiatric individuals [3,4]. In children, coins and button batteries are the commonly ingested foreign bodies, whereas, in adults, dentures are most common [1,5]. This is because dentures cause a gradual loss of sensation of the oral cavity and laryngopharynx. Besides, there is also an increase in the denture-wearing population that leads to an increase in the incidence of denture ingestion [2].

The most common presenting symptom following the accidental swallowing of a denture is dysphagia [6]. The other symptoms include hypersalivation, retrosternal fullness, and regurgitation of foods. The incidence and type of complications correlate with the site and duration of the impaction. If a denture gets impacted in the mid-esophagus, the incidence of complications is quite high, owing to its proximity to the anatomical structures [7]. If it gets impacted for a long period, it will cause mucosal ulceration, perforation, para or retro-esophageal abscess, mediastinitis, empyema, or even tracheo and aorta-esophageal fistula [3,5]. But it is rare for a foreign body to remain in the esophagus for a long period without any complications. There was nothing peculiar about the denture characteristics, nor did the patient have any psychiatric illness to remain asymptomatic for such a long time. After a thorough review of the literature, we found some long-lasting esophageal foreign bodies in children, but only two reported in adults (Table 1) [8]. The denture was lodged in the esophagus for less than a year in both these cases. But, in our case, the denture remained asymptomatic in the mid-esophagus for more than a decade.

**Table 1:** Summary of the articles published about a long-lasting esophageal foreign body in an adult

Author	Year	Patient age/sex	Type of foreign body	Site of lodgement	Duration of lodgement	Treatment
Kropf JA et al. [9]	2013	82/F	Endoscope capsule	Zenker diverticulum	Four months	Removal by laryngoscopy
Mohajeri et al. [6]	2015	57/M	Denture	Mid-esophagus	Nine months	Mini-laparotomy and gastrotomy
Our case	2020	45/F	Denture	Mid-esophagus	13 years	Thoracotomy and esophagotomy

A soft tissue neck and chest radiograph is the initial investigation of choice. Dentures, however, are frequently made of acrylic resin, which is a radiolucent material, and they are difficult to assess on plain X-rays. But the radio-opaque wire hooks of the dentures can be seen [5]. It is difficult to assess the relationship between the impacted denture and the surrounding tissue with an X-ray. An unenhanced CT scan has 100% sensitivity, 92.6% specificity, 97.9% positive predictive value, and 100% negative predictive value in the diagnosis of the esophageal foreign bodies [7,10]. Hence, CT scan is a gold standard for identifying the foreign body location and its associated complications and thereby helps the surgeon in preoperative planning [7].

The management strategies for the removal of the esophageal foreign body include endoscopic removal and surgery. Attempts at endoscopic removal of the impacted dentures may cause intramural perforation or a full-thickness tear owing to the possible entrapment of wire hooks in the esophageal wall [1]. Therefore, surgery remains a safe and effective treatment for patients with impacted dentures in the esophagus.

## Conclusions

To the best of our knowledge, this is the first case in the literature reporting a foreign body that remained asymptomatic in the esophagus for more than a decade. Endoscopic removal of long-standing impacted dentures will cause extensive laceration of the esophagus and is therefore contraindicated. Surgery remains a safe and effective treatment for patients with such long-standing impacted dentures in the esophagus.

## References

[1] Mohanty HS, Shirodkar K, Patil AR, Mallarajapatna G, Kumar S, Deepak KC, Nandikoor S (2016). Oesophageal perforation as a complication of ingested partial denture. BJR.

[2] Thapar VK, Jagtap S, Barve DJ, Savarkar DP, Garle MN, Shukla AP (2010). Thoracoscopic removal of impacted denture: report of a case with review of literature. J Min Access Surg.

[3] Mughal Z, Charlton AR, Dwivedi R, Natesh B (2019). Impacted denture in the oesophagus: review of the literature and its management. BMJ Case Rep.

[4] Dörner J, Spelter H, Zirngibl H, Ambe PC (2017). Surgical retrieval of a swallowed denture in a schizophrenic patient: a case report. Patient Saf Surgery.

[5] Singh P, Singh A, Kant P, Zonunsanga B, Kuka AS (2013). An impacted denture in the oesophagus- an endoscopic or a surgical emergency-a case report. J Clin Diagn Res.

[6] Mohajeri G, Fakhari S, Ghaffarzadeh Z, Piri-Ardakani M (2016). A case of the long time presence of a large foreign body in esophagus without complication. Adv Biomed Res.

[7] Wang F, Yang N, Wang Z, Guo X, Hui L (2019). Clinical analysis of denture impaction in the esophagus of adults. Dysphagia.

[8] Miller RS, Willging JP, Rutter MJ, Rookkapan K (2004). Chronic esophageal foreign bodies in pediatric patients: a retrospective review. Int J Pediatr Otorhinolaryngol.

[9] Kropf JA, Jeanmonod R, Yen DM (2013). An unusual presentation of a chronic ingested foreign body in an adult. J Emerg Med.

[10] Liu YC, Zhou SH, Ling L (2013). Value of helical computed tomography in the early diagnosis of esophageal foreign bodies in adults. Am J Emerg Med.

